# Structural, Thermal, and Phenol Adsorption Properties of a Humic Acid/Reduced Graphene Oxide Composite

**DOI:** 10.3390/ma19163371

**Published:** 2026-08-07

**Authors:** Alma Khassenovna Zhakina, Oxana Vasilievna Arnt, Yevgeniy Petrovich Vassilets, Almat Maulenuly Zhakin, Zainulla Muldakhmetov

**Affiliations:** 1 LLP “Institute of Organic Synthesis and Coal Chemistry of the Republic of Kazakhstan”, Karaganda 100008, Kazakhstan; oxana230590@mail.ru (O.V.A.); vassilets88@mail.ru (Y.P.V.); zhakin-almat@mail.ru (A.M.Z.); iosu.rk@mail.ru (Z.M.); 2 Department of Chemical Technology and Ecology, Faculty of Metallurgy and Mechanical Engineering, Karaganda Industrial University, Temirtau 101400, Kazakhstan

**Keywords:** ultrasonic treatment, oxygen-containing functional groups, interfacial interactions, supramolecular organization, carbonaceous sorbent, water purification

## Abstract

A composite based on humic acid (HA) and reduced graphene oxide (rGO) was synthesized to evaluate the effect of rGO on the structural, functional, thermal, and preliminary phenol adsorption properties of humic acid. The incorporation of rGO increased the carbon content from 47.34 to 55.61 wt.% and decreased the oxygen content from 48.31 to 40.79 wt.%. At the same time, the total content of carboxyl and phenolic hydroxyl groups increased from 5.00 to 5.47 mmol/g, indicating improved accessibility of oxygen-containing functional sites. FTIR spectroscopy confirmed the retention of the main functional groups of the initial components after composite formation. Thermogravimetric analysis showed enhanced thermal stability, with the residual mass at 1000 °C increasing from 59.21 to 70.03%. Electron microscopy revealed the formation of a developed wrinkled surface morphology. Preliminary phenol adsorption experiments showed that the HA-rGO composite exhibited higher adsorption capacity than the initial HA and rGO. This improvement was attributed to the combined contribution of oxygen-containing functional groups and the aromatic carbon structure of rGO, which may promote hydrogen bonding and π–π interactions with phenol molecules.

## 1. Introduction

Humic acids (HA) are natural high-molecular-weight organic compounds formed during the humification of plant residues and are among the major components of organic matter in soils, peat, and coals. Their structure includes aromatic and aliphatic fragments bearing carboxyl, phenolic, hydroxyl, and carbonyl functional groups. The presence of a developed system of reactive sites determines the ability of humic acids to participate in complexation, ion exchange, and the formation of various hybrid materials [1,2,3,4].

In recent years, humic acids have been considered not only as natural organic substances but also as promising feedstocks for the preparation of functional materials for environmental, sorption, catalytic, and technological applications. Their main advantages include the availability of raw-material sources, high chemical activity, and the possibility of directed structural modification. However, the practical use of humic acids is often limited by their relatively low thermal stability, tendency to aggregate, and insufficiently developed surface [4,5,6,7].

One effective approach to modifying humic acids is their combination with carbon nanomaterials. Among these materials, reduced graphene oxide (rGO) is of particular interest because of its high degree of aromatization, developed surface, chemical stability, and system of conjugated π-electrons. Owing to these properties, rGO can interact effectively with organic macromolecules and participate in the formation of hybrid composite structures [8,9,10,11,12,13,14,15,16].

The incorporation of graphene materials into various organic matrices can modify the surface morphology, increase thermal stability, improve structural organization, and impart new functional properties to composites. In most cases, interactions between the components are governed by non-covalent forces, including hydrogen bonding and π–π interactions between aromatic fragments [14,15,16,17,18].

The literature reports composites based on humic substances and different carbon materials, including graphene oxide, carbon nanotubes, biochar, and activated carbon materials. The incorporation of carbon nanostructures has been shown to change surface morphology, increase resistance to thermal exposure, and improve several functional characteristics of the resulting materials [19,20,21,22,23,24,25,26,27].

However, information on composites based on humic acid and reduced graphene oxide remains limited. Most published studies focus on the applied properties of related materials, whereas the features of their formation and changes in functional composition, surface morphology, and thermal behavior have not been examined in sufficient detail. In addition, systematic data on the relationship between elemental composition, functional-group content, surface morphology, and thermal stability of HA-rGO composites are still lacking [19,20,23].

Previous studies involving humic acid and graphene-related materials have mainly focused on the production of porous carbon materials through high-temperature carbonization or chemical reduction in HA–GO precursors, as well as on the conversion of humic acid into graphene-like materials by hydrothermal treatment. In contrast, the present work examines a non-carbonized HA–rGO composite prepared directly in an aqueous medium using ultrasonic treatment, with humic acid retained as the predominant matrix component. The study does not claim the first preparation of a humic acid/graphene-based composite. Its scientific contribution lies in the integrated evaluation of how rGO incorporation affects the elemental composition, accessibility of oxygen-containing functional groups, surface morphology, thermal stability and preliminary phenol adsorption behavior within the same material system.

Accordingly, the novelty of this study lies not in the first synthesis of an HA/graphene-based material, but in establishing, within a non-carbonized ultrasound-assisted HA–rGO system, the relationship between changes in elemental and functional composition, surface morphology, thermal behavior and phenol adsorption performance.

The aim of this work was to determine the effect of reduced graphene oxide on the elemental composition, content of oxygen-containing functional groups, surface morphology, and thermal stability of humic acid during the formation of an HA-rGO composite, as well as to preliminarily evaluate its adsorption behavior toward phenol as a model aromatic pollutant.

To achieve this aim, the elemental composition and functional-group content were determined, and FTIR spectroscopy, thermogravimetric analysis, scanning electron microscopy, and energy-dispersive X-ray spectroscopy were performed for the synthesized composite and the initial components.

## 2. Materials and Methods

### 2.1. Materials

Humic acid, isolated from oxidized coal from the Shubarkol deposit (Karaganda, Kazakhstan) and converted into the acid form according to a previously developed procedure, was used as the main component [28]. Humic acid is a natural polyfunctional organic material containing a wide range of oxygen-containing functional groups, including carboxyl, phenolic, hydroxyl, and carbonyl fragments capable of participating in various intermolecular interactions [29,30,31,32,33].

Reduced graphene oxide from Sigma-Aldrich (St. Louis, MO, USA) was used as the modifying component. Reduced graphene oxide is a highly dispersed carbon material with a developed surface and a significant amount of residual oxygen-containing functional groups [10,11,14,15,16,17,18].

Distilled water was used for the preparation of all solutions. The pH was adjusted using analytical-grade 0.1 M NaOH and 0.1 M HCl solutions. Adsorption studies were carried out using freshly prepared aqueous phenol solutions with initial concentrations ranging from 0.5 to 15 mg/dm^3^.

### 2.2. Synthesis of the HA-rGO Composite

The composite was synthesized by ultrasonic treatment in an aqueous medium.

In the first step, humic acid was dispersed in distilled water under continuous stirring until a homogeneous suspension was obtained. To increase the solubility of humic substances and improve the accessibility of functional groups, the pH of the medium was adjusted to 9 by adding sodium hydroxide solution. After stabilization of the system, reduced graphene oxide was introduced into the suspension. The HA:rGO mass ratio was 80:20. The initial solutions of HA and the dispersion of rGO were mixed with constant stirring to ensure a uniform distribution of the components in the volume of the reaction mixture. The HA = 80:20 ratio was chosen as a working compound that provides sufficient rGO content for the formation of a composite structure and its effect on the morphological, thermal and adsorption properties of the material while maintaining humic acid as the main component of the matrix. Ultrasonic treatment was used to intensify interactions between the components and ensure a uniform distribution of the graphene material within the humic matrix. Treatment was carried out using a SCIENTZ-650E ultrasonic homogenizer (Ningbo Scientz Biotechnology Co., Ltd., Ningbo, China) at a frequency of 20–25 kHz and a power of 60 W for 30 min. Ultrasonic exposure promotes system homogenization and increases the contact area between humic acid and reduced graphene oxide [34,35,36,37].

After ultrasonic treatment, the suspension was kept until completion of structure formation, after which the obtained material was dried to constant weight. The dried product was ground and used for subsequent physicochemical studies.

### 2.3. Elemental Analysis and Determination of Functional Groups

The elemental composition of the samples was determined using an Elementar Unicube elemental analyzer (Elementar Analysensysteme GmbH, Langenselbold, Germany). The contents of carbon, hydrogen, and oxygen were calculated according to the standard procedures provided by the instrument software.

To quantitatively evaluate the degree of oxidation and aromatization of the materials, the atomic oxygen-to-carbon ratio (O/C) was calculated from elemental-analysis data using the following equation:

(1) $$O/C=\frac{\left(\frac{O\%}{16}\right)}{\left(\frac{C\%}{12}\right)},$$

where *O*% and *C*% are the mass fractions of oxygen and carbon, respectively. This parameter is widely used to characterize carbon materials, including humic substances and graphene-like structures, and provides an indirect estimate of the content of oxygen-containing functional groups and the degree of aromatization of the carbon framework [1,2,3,10,11,14].

The content of oxygen-containing functional groups was determined by selective chemical binding followed by titrimetric analysis. Sodium acetate was used to determine carboxyl groups, while the total content of carboxyl and phenolic hydroxyl groups was determined by treating the samples with barium hydroxide solution. After equilibrium was reached, the excess reagent was titrated with a standard hydrochloric acid solution. The functional-group content was calculated in mmol/g of absolutely dry substance.

### 2.4. Fourier Transform Infrared Spectroscopy

The functional groups in the samples were identified by Fourier transform infrared (FTIR) spectroscopy. Spectra were recorded on an FSM-1201 FTIR spectrometer (Infraspek, St. Petersburg, Russia) in the wavenumber range of 400–4000 cm^−1^.

Samples were prepared by pressing with potassium bromide (KBr). The obtained spectra were mathematically processed and analyzed using Fityk 1.3.1 software. Absorption bands were assigned on the basis of literature data and known characteristic vibration regions of functional groups [38,39].

### 2.5. Thermogravimetric and Differential Thermal Analysis

The thermal stability of the initial components and synthesized composites was examined by simultaneous thermogravimetric and differential thermal analysis using an STA 6000 instrument (PerkinElmer, Shelton, CT, USA).

Measurements were performed in an argon atmosphere at a gas flow rate of 50 mL/min over the temperature range from room temperature to 1000 °C. The heating rate was 10 °C/min. Corundum crucibles (Al_2_O_3_) were used as sample containers. The obtained TG, DTG, and DTA curves were used to evaluate the stages of thermal decomposition and the thermal stability of the materials.

### 2.6. Scanning Electron Microscopy and Energy-Dispersive Analysis

Surface morphology was studied by scanning electron microscopy using a MIRA 3 microscope (TESCAN, Brno, Czech Republic). Before analysis, the samples were deposited on a conductive substrate and coated with a thin gold layer to prevent surface charging under the electron beam.

The measurements were performed at an accelerating voltage of 5 kV, an electron-beam current of 86 pA, and a working distance of 3.3 mm. Images were recorded in secondary-electron mode at magnifications up to ×50,000.

The elemental composition and distribution of elements over the sample surface were determined by energy-dispersive X-ray spectroscopy (EDS) (TESCAN, Brno, Czech Republic) integrated into the scanning electron microscope system. The obtained data were used to construct elemental distribution maps and to assess the homogeneity of the composite structure.

### 2.7. Phenol Adsorption Experiments

Phenol adsorption was evaluated as a preliminary functional test of the synthesized HA-rGO composite. The experiments were performed under static batch conditions at 25 °C using freshly prepared aqueous phenol solutions with initial concentrations ranging from 0.5 to 15 mg/dm^3^. The sorbent-to-solution ratio was 0.1 g per 100 mL. After adsorption, the solid phase was separated by filtration, and the residual phenol concentration in the filtrate was determined. Phenol was quantified using a Fluorat-02-2M liquid analyzer (Lumex, St. Petersburg, Russia) according to ST RK 2359-2013 [40], which is based on the fluorimetric determination of phenolic compounds after extraction and re-extraction steps. All adsorption experiments were performed in triplicate, and the results are presented as mean values ± standard deviation. The error bars represent the standard deviations of three independent measurements. The adsorption capacity *q_e_* (mg/g) and removal efficiency *J* (%) were calculated using the following equations:

(2) $$q_{e}=\frac{\left(C_{0}-C_{e}\right)V}{m}$$

(3) $$J=\frac{C_{0}-C_{e}}{C_{0}}\cdot 100\%$$

where *C*_0_ and *C_e_* are the initial and equilibrium phenol concentrations, respectively (mg/dm^3^); V is the solution volume (dm^3^); and m is the sorbent mass (g).

The equilibrium adsorption data were analyzed using the Langmuir and Freundlich isotherm models. The linear form of the Langmuir equation was expressed as:

(4) $$\frac{C_{e}}{q_{e}}=\frac{1}{q_{m}K_{L}}+\frac{C_{e}}{q_{m}}$$

where *q_m_* is the maximum monolayer adsorption capacity (mg/g), and *K_L_* is the Langmuir equilibrium constant (dm^3^/mg).

The linear form of the Freundlich equation was expressed as:

(5) $$lg\;q_{e}=lg\;K_{F}+\frac{1}{n}lgC_{e}$$

where *K_F_* is the Freundlich adsorption constant and *n* is the adsorption-intensity parameter.

Kinetic experiments were performed at an initial phenol concentration of 7.5 mg/dm^3^. The kinetic data were analyzed using the pseudo-first-order and pseudo-second-order models. The linear forms of these equations were expressed as:

(6) $$ln\left(q_{e}-q_{t}\right)=lnq_{e}-k_{1}t,$$

(7) $$\frac{t}{q_{t}}=\frac{1}{k_{2}q_{e}^{2}}+\frac{t}{q_{e}},$$

where *q_t_* is the adsorption capacity at time *t* (mg/g), *k*_1_ is the pseudo-first-order rate constant (min^−1^), and *k*_2_ is the pseudo-second-order rate constant (g·mg^−1^·min^−1^). Model parameters were determined by linear regression, and the goodness of fit was evaluated using the coefficient of determination R^2^.

## 3. Results and Discussion

### 3.1. Elemental Composition and Content of Oxygen-Containing Functional Groups

The results of elemental analysis, determination of the total content of oxygen-containing functional groups Σ(COOH + OH), and yield of the synthesized composite are presented in Table 1.

As shown in Table 1, the initial humic acid is characterized by a high oxygen content (48.31 wt.%) and a relatively low carbon content (47.34 wt.%), which is due to the large amount of oxygen-containing functional groups present in its structure. Humic acids are known to be complex natural polyfunctional macromolecular systems comprising aromatic and aliphatic fragments bearing carboxyl, phenolic, alcoholic, and carbonyl groups. These functional centers determine the high chemical activity of humic substances and their ability to interact with various organic and inorganic compounds [1,2,3,4].

In contrast to humic acid, reduced graphene oxide contains considerably more carbon (67.49 wt.%) and less oxygen (31.94 wt.%). These results indicate substantial removal of oxygen-containing groups compared with graphene oxide and the formation of a more condensed aromatic carbon structure. The low hydrogen content (0.57 wt.%) further confirms the high degree of aromatization and graphitization of reduced graphene oxide [10,11,14,15,16,17,18].

The synthesized HA-rGO composite shows intermediate elemental-composition values. The carbon content increases to 55.61 wt.%, whereas the oxygen content decreases to 40.79 wt.%. This change in composition indicates the successful incorporation of reduced graphene oxide into the humic matrix and the formation of a hybrid carbon-containing material. At the same time, the decrease in hydrogen content to 3.60 wt.% indicates an increased fraction of the aromatized carbon phase in the composite structure. The incorporation of reduced graphene oxide therefore increases the degree of aromatization of the material, as reflected by the increase in carbon content from 47.34 to 55.61 wt.% and the simultaneous decrease in oxygen content from 48.31 to 40.79 wt.%. Such a change in elemental composition is characteristic of hybrid carbon-containing materials based on humic substances and graphene-like structures [19,20,23].

The calculated atomic O/C ratio for the initial humic acid is 0.77, which is typical of highly oxidized natural organic materials. For reduced graphene oxide, this value is expectedly lower (0.36), reflecting the high degree of reduction and aromatization of its structure.

For the HA-rGO composite, the O/C ratio decreases to 0.73. Although this change appears modest, it should be noted that, upon the addition of 20 wt.% rGO with a low oxygen content (31.94 wt.%), a simple mechanical mixture would be expected to have an O/C ratio of approximately 0.66 (calculated value). Thus, the experimentally observed value of 0.73 is considerably higher, indicating effective retention of the oxygen-containing functional groups of humic acid within the composite structure. This result agrees with the increase in the total content of carboxyl and phenolic groups (5.00 → 5.47 mmol/g) and suggests that interaction with rGO promotes not destruction of the humic matrix but its disaggregation, exposing additional active sites.

The results for the total content of oxygen-containing functional groups, Σ(COOH + OH), are of particular interest. Despite the lower oxygen content in reduced graphene oxide, the HA-rGO composite shows an increase in Σ(COOH + OH) to 5.47 mmol/g compared with the initial humic acid (5.00 mmol/g). This result indicates that the incorporation of reduced graphene oxide does not decrease the number of accessible functional groups; rather, it increases their accessibility for interaction with the titrant.

A probable reason for this effect is structural rearrangement of the humic matrix during ultrasonic treatment. Ultrasonic exposure likely promotes partial disaggregation of supramolecular humic acid associates, thereby increasing the accessibility of some carboxyl and phenolic groups to the titrant. Interactions between the aromatic fragments of humic acid and the graphene-like layers of reduced graphene oxide may also contribute to this effect [15,16,21].

It should be noted that an increase in accessible oxygen-containing groups together with an increase in the carbon fraction is of particular interest for the further use of the composite as a functional carbon material. This combination enables the retention of a high concentration of active sites while simultaneously increasing the degree of structural aromatization.

The yield of the HA-rGO composite was 87.13%, which is substantially higher than that of the initial humic acid (70.05%). The high product yield indicates effective incorporation of reduced graphene oxide into the composite and confirms the efficiency of the selected ultrasound-assisted synthesis method. Taken together, the obtained data indicate the formation of a new hybrid material combining the developed system of oxygen-containing functional groups of humic acid with the high degree of aromatization of reduced graphene oxide [19,20,23].

### 3.2. Analysis of FTIR Spectra of the Initial Components and the HA-rGO Composite

**Figure 1:**
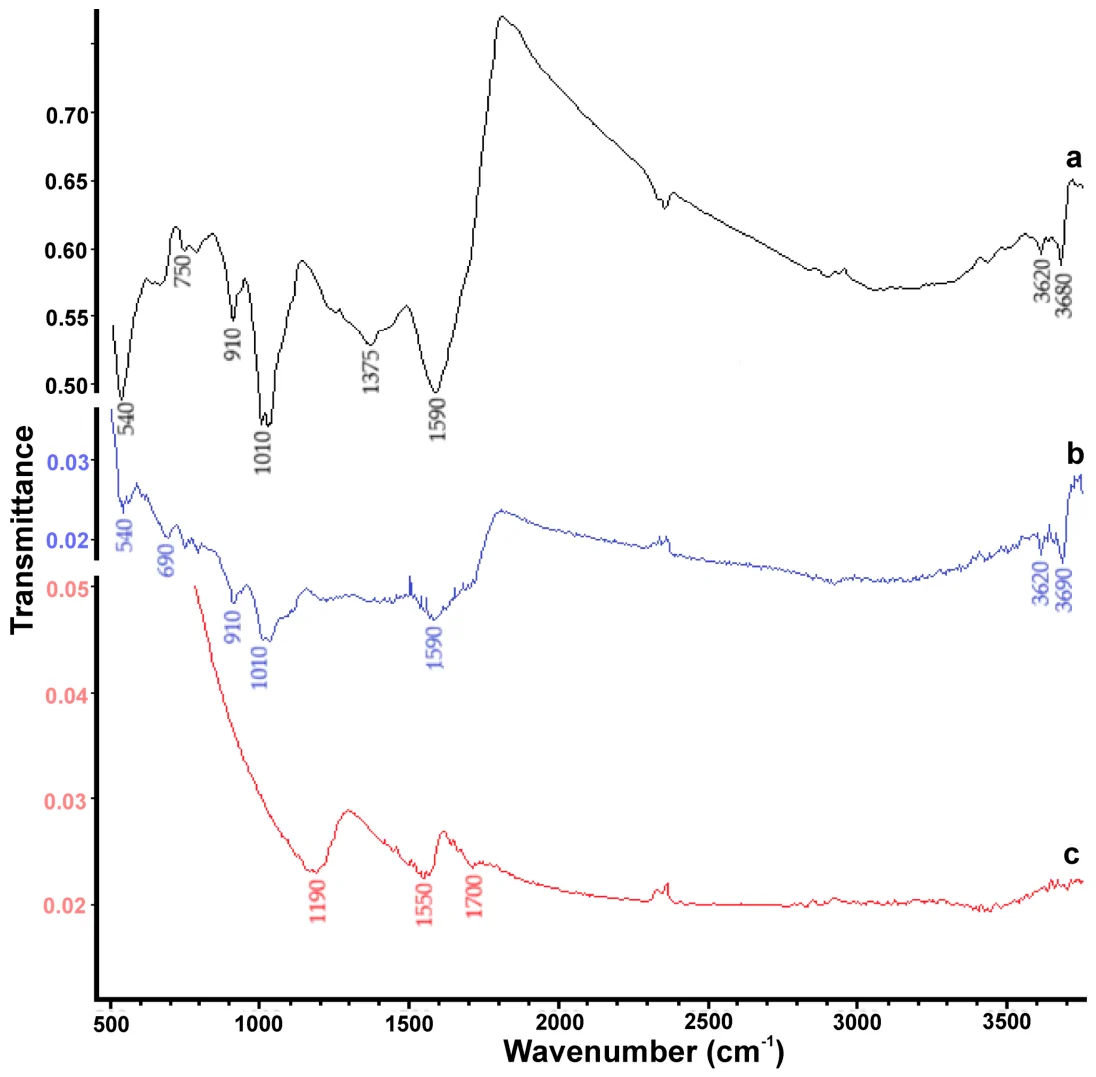
FTIR spectra of humic acid (**a**), HA-rGO composite (**b**), and reduced graphene oxide (**c**).

FTIR spectroscopy was used to study the functional composition of the initial humic acid, reduced graphene oxide, and synthesized HA-rGO composite. The obtained spectra are shown in Figure 1.

As shown in Figure 1a, the spectrum of the initial humic acid contains a broad intense absorption band at 3600–3200 cm^−1^, corresponding to O–H stretching vibrations of hydroxyl groups in phenolic, alcoholic, and carboxylic fragments. The presence of this band indicates a high degree of humic acid functionalization by oxygen-containing groups [38,39].

An intense absorption band is observed at approximately 1590 cm^−1^ and is associated with vibrations of aromatic C=C bonds, as well as the contribution of asymmetric vibrations of carboxylate groups. This band is characteristic of humic substances and reflects the presence of aromatic structural fragments in the macromolecular matrix [38,39].

The band at 1375 cm^−1^ can be assigned to deformation vibrations of hydroxyl groups and C–H vibrations of aliphatic fragments. Intense absorption at approximately 1010 cm^−1^ is related to C–O stretching vibrations of phenolic, alcoholic, and ether groups. Additional bands in the 910–700 cm^−1^ region are associated with vibrations of aromatic structures and oxygen-containing functional groups [38,39].

The spectrum of reduced graphene oxide (Figure 1c) differs substantially from that of humic acid. For rGO, an absorption band is observed at approximately 1700 cm^−1^, corresponding to C=O stretching vibrations of carbonyl groups retained after reduction. The band at approximately 1550 cm^−1^ is associated with vibrations of the aromatic carbon framework characteristic of graphene-like materials. Absorption at 1190 cm^−1^ is attributed to vibrations of C–O and C–O–C bonds of residual oxygen-containing groups [10,11,41].

The lower intensity of bands associated with hydroxyl and oxygen-containing groups, compared with humic acid, confirms the higher degree of aromatization and condensation of the reduced graphene oxide structure.

The spectrum of the HA-rGO composite (Figure 1b) combines features of both initial components. The main absorption bands characteristic of humic acid are retained, including the hydroxyl-group band at 3600–3200 cm^−1^, aromatic-structure signals around 1590 cm^−1^, and the C–O stretching band at 1010 cm^−1^. At the same time, bands characteristic of reduced graphene oxide also contribute to the spectrum, indicating successful formation of the composite structure.

The retention of the band in the 1590–1550 cm^−1^ region, corresponding to vibrations of aromatic structures, is especially important. This observation indicates that the aromatic framework of both components is preserved after ultrasonic treatment. No substantial shifts in the positions of the main absorption bands are observed, indicating retention of the functional structure of the initial components after composite synthesis.

No new intense absorption bands are observed in the composite spectrum that would indicate the formation of new covalent bonds between the components. This suggests that the interaction between humic acid and reduced graphene oxide is governed mainly by non-covalent interactions. The most probable interactions include hydrogen bonding between oxygen-containing functional groups and π–π interactions between aromatic fragments of the components. These interactions may promote the formation of a stable interfacial boundary and facilitate the integration of graphene structures into the humic matrix. Thus, the integration of the components can be attributed to a combination of hydrogen bonding, π–π stacking of aromatic fragments, and intermolecular interactions between the oxygen-containing groups of humic acid and residual functional sites on the surface of reduced graphene oxide [15,16,21].

Thus, FTIR spectroscopy confirms the retention of the main functional groups of humic acid after composite synthesis and indicates the formation of a hybrid structure through intermolecular interactions between the components without the formation of new covalent bonds.

### 3.3. Thermal Properties of Humic Acid, Reduced Graphene Oxide, and the HA-rGO Composite

**Figure 2:**
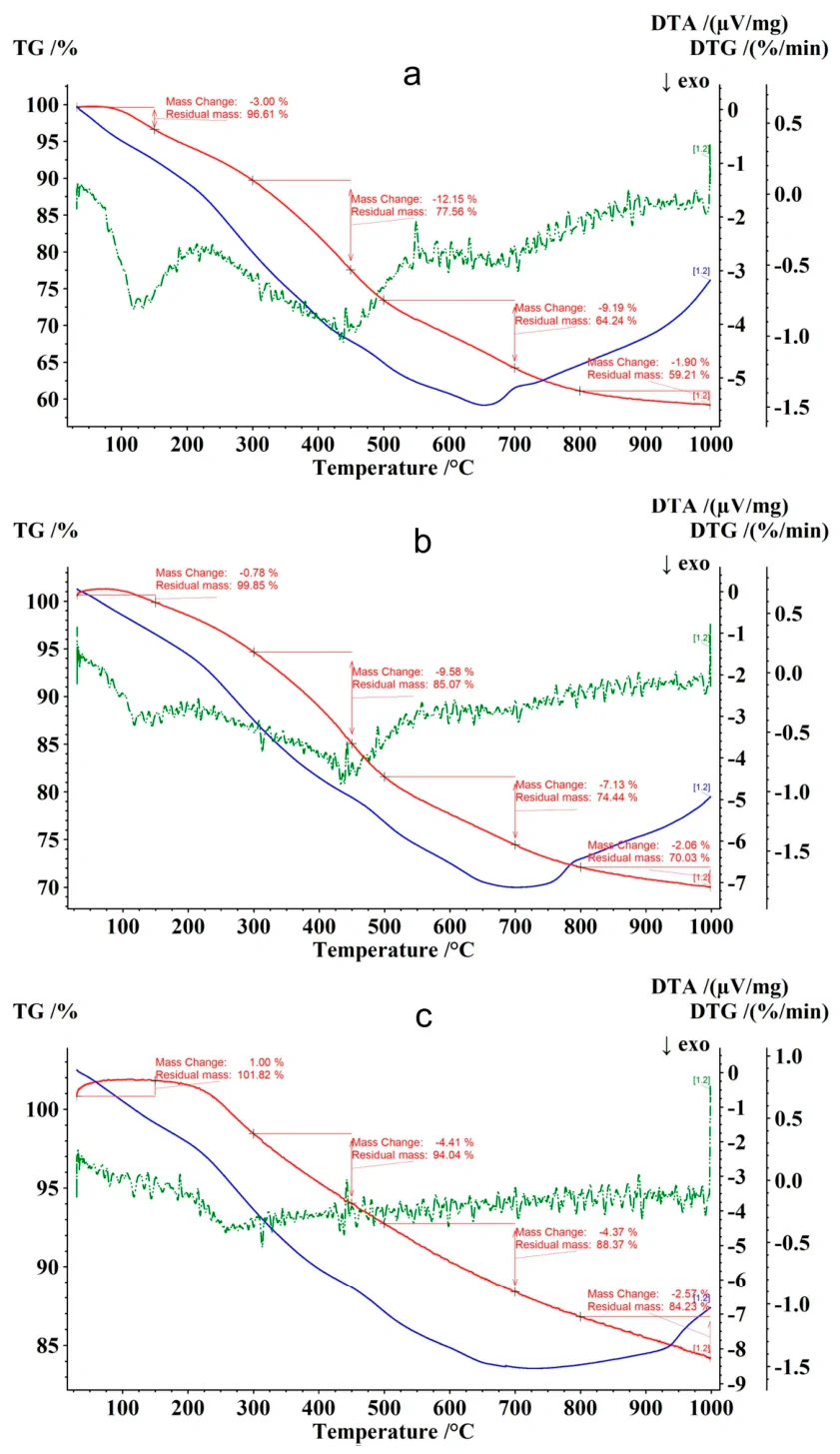
TG, DTG, and DTA curves of the studied samples: (**a**) humic acid, (**b**) HA-rGO composite, and (**c**) reduced graphene oxide.

The thermal stability of the initial humic acid, reduced graphene oxide, and HA-rGO composite was investigated by thermogravimetric analysis over the temperature range of 30–1000 °C in an argon atmosphere. The obtained TG, DTG, and DTA curves are presented in Figure 2a–c.

For quantitative evaluation of thermal decomposition, mass losses in individual temperature intervals were calculated (Table 2).

As shown in Figure 2a–c and Table 2, thermal decomposition of all samples proceeds through a multistage mechanism. The first stage, observed in the 30–150 °C range, is mainly associated with removal of physically adsorbed moisture and low-molecular-weight volatile compounds. The largest mass loss at this stage is observed for humic acid (3.00%), which is due to its high content of hydrophilic oxygen-containing functional groups. For the HA-rGO composite and reduced graphene oxide, the corresponding mass losses are considerably lower, 0.78% and 1.00%, respectively [20,42].

The main degradation of the organic matrix occurs in the 150–450 °C range. For humic acid, the maximum mass loss in this interval is 12.15%, which is associated with decomposition of carboxyl, phenolic, and other oxygen-containing groups. After the incorporation of reduced graphene oxide, the mass loss decreases to 9.58%, indicating increased resistance of the composite structure to thermal exposure. For reduced graphene oxide, the mass loss is only 4.41%, which is associated with the high degree of aromatization of its carbon framework [42,43].

In the 450–700 °C range, destruction of more stable organic fragments and further carbonization of the material continue. The mass loss of humic acid at this stage is 9.19%, whereas that of the HA-rGO composite decreases to 7.13%. For reduced graphene oxide, the mass loss remains minimal (4.37%), further confirming the high thermal stability of the graphene structure [42,43].

Above 700 °C, mass changes in all samples become less pronounced and are mainly associated with the completion of carbonization processes. The mass losses in this interval do not exceed 3%.

The most pronounced differences between the samples are observed in the residual mass at 1000 °C. The residual mass of the initial humic acid is 59.21%, whereas that of the HA-rGO composite increases to 70.03%. Thus, the incorporation of reduced graphene oxide increases the carbon residue yield by almost 11%. The improved thermal stability of the composite is consistent with the decrease in the atomic O/C ratio from 0.77 for humic acid to 0.73 for the composite. The decrease in oxygen fraction and increase in carbon content indicate the formation of a more aromatized structure resistant to thermal degradation. The highest thermal stability is observed for reduced graphene oxide, whose residual mass reaches 84.23%. It should be noted that the increase in the residual mass of the composite is due not only to the presence of a thermally stable graphene component but also to lower mass losses in the 150–700 °C interval. This fact indicates a change in the thermal-degradation mechanism of the humic matrix after the incorporation of reduced graphene oxide and the formation of a more stable carbon structure [10,11,20,23,42,43].

Analysis of the DTG curves shows that the most intense decomposition processes occur in the 400–500 °C region. For the HA-rGO composite, the intensity of mass loss is noticeably lower than that of the initial humic acid. At the same time, the DTA curves show less pronounced thermal effects associated with degradation of the organic matrix, which also indicates the stabilizing effect of the graphene component.

The increase in thermal stability of the composite may be related to the formation of intermolecular interactions between humic acid and reduced graphene oxide. Interactions between aromatic fragments of humic acid and the graphene-like layers of rGO via π–π stacking likely promote the formation of a more condensed structure resistant to thermal exposure. The high intrinsic thermal stability of reduced graphene oxide may also contribute to this effect [10,11,17,44].

Thus, thermogravimetric analysis shows that the incorporation of reduced graphene oxide substantially improves the thermal stability of humic acid, as confirmed by lower mass losses at the main decomposition stages and an increase in residual mass at 1000 °C from 59.21 to 70.03%. This indicates the formation of a more stable carbon structure within the HA-rGO composite.

### 3.4. Surface Morphology According to Scanning Electron Microscopy

**Figure 3:**
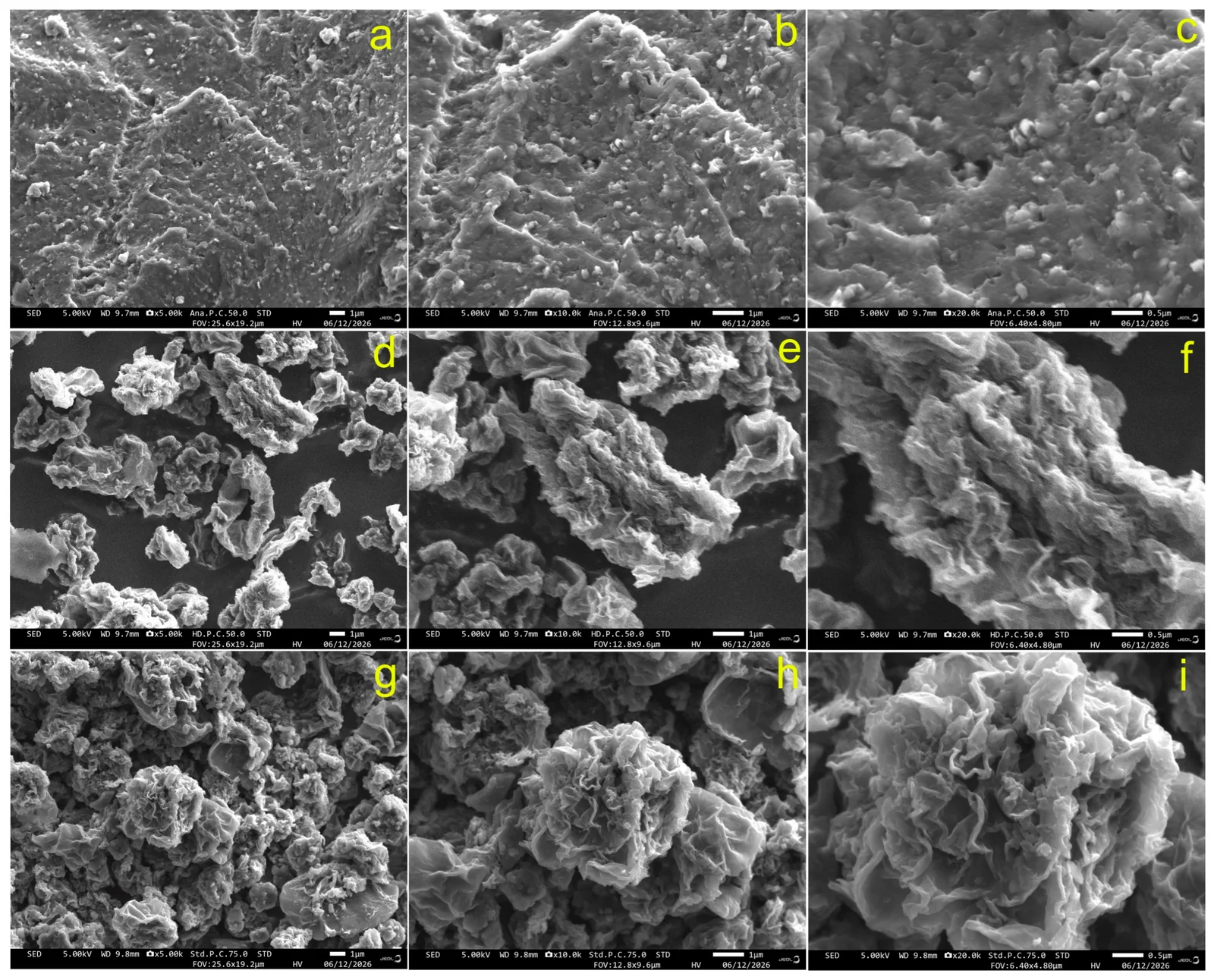
SEM images of the studied samples at magnifications of ×5000, ×10,000, and ×20,000: (**a**–**c**) HA; (**d**–**f**) rGO; and (**g**–**i**) HA-rGO.

The morphological features of the initial humic acid, reduced graphene oxide, and HA-rGO composite were studied by scanning electron microscopy. The obtained micrographs are shown in Figure 3a–i.

The initial humic acid is characterized by a dense aggregated structure with a relatively homogeneous surface (Figure 3a–c). At ×5000 magnification, large massive irregularly shaped formations are observed, forming a continuous surface without pronounced layered organization. Increasing the magnification to ×10,000 and ×20,000 reveals a developed microrelief represented by numerous protrusions, depressions, and individual pores of micron and submicron size. The HA surface is relatively compact and is characterized by a high degree of particle aggregation, which is associated with the complex supramolecular organization of humic substances [1,2,3].

The morphology of reduced graphene oxide differs substantially from that of humic acid (Figure 3d–f). rGO consists of thin crumpled sheet-like formations that form loose aggregates of different sizes. The micrographs clearly show folds, bends, and wavy surface regions characteristic of graphene-like materials. At ×20,000 magnification, individual multilayer structures formed by overlapping and partial folding of graphene sheets are clearly distinguishable. Free spaces between individual aggregates form a developed open structure [44,45,46,47].

After composite formation, substantial changes in surface morphology are observed (Figure 3g–i). Unlike the dense structure of the initial humic acid and the separate sheet-like formations of reduced graphene oxide, the composite is characterized by the formation of large aggregates with complex shapes. Wrinkled structures resembling crumpled and partially folded sheet-like formations are clearly visible on the material surface. The rounded pompon-like features observed in the HA-rGO micrographs should not be interpreted as individual spherical particles. They represent three-dimensional aggregates assembled from numerous wrinkled and partially folded sheet-like fragments. Their occurrence at different magnifications indicates that they are characteristic morphological elements of the examined composite regions, although their quantitative abundance was not determined.

At ×5000 magnification, the composite appears as a system of three-dimensional aggregates with a developed rough surface. At ×10,000 and ×20,000 magnifications, the aggregates are seen to consist of numerous thin wrinkled fragments forming a spatial structure with a large number of cavities and interparticle voids. Compared with the initial humic acid, the composite surface becomes more developed and heterogeneous. The observed wrinkled morphology contains visible interparticle cavities and structural irregularities that may influence surface accessibility. However, their contribution to the specific surface area, pore structure and adsorption capacity cannot be quantified from SEM observations alone. Such structural organization may affect the accessibility of humic acid functional groups; however, this interpretation requires confirmation by quantitative surface-area and pore-structure measurements.

It should be noted that the composite does not contain large areas of dense humic matrix typical of the initial HA, nor separate free graphene sheets typical of rGO. This indicates a substantial change in the morphology of both components during ultrasonic treatment and subsequent formation of the composite material.

The observed structure indicates effective interaction between humic acid and the surface of reduced graphene oxide. During synthesis, humic acid macromolecules probably adsorb onto the surface of graphene sheets, leading to their partial aggregation and the formation of larger wrinkled structures. As a result, a single hybrid structure is formed, with a morphology that differs substantially from those of the initial components [19,20,44,45,46,47].

Thus, scanning electron microscopy confirms the formation of a new composite material based on humic acid and reduced graphene oxide. The formation of large wrinkled aggregates, the absence of free graphene sheets, and the change in surface character compared with the initial components indicate structural interaction between the composite components during synthesis.

### 3.5. Energy-Dispersive X-Ray Spectroscopy and Elemental Mapping

**Figure 4:**
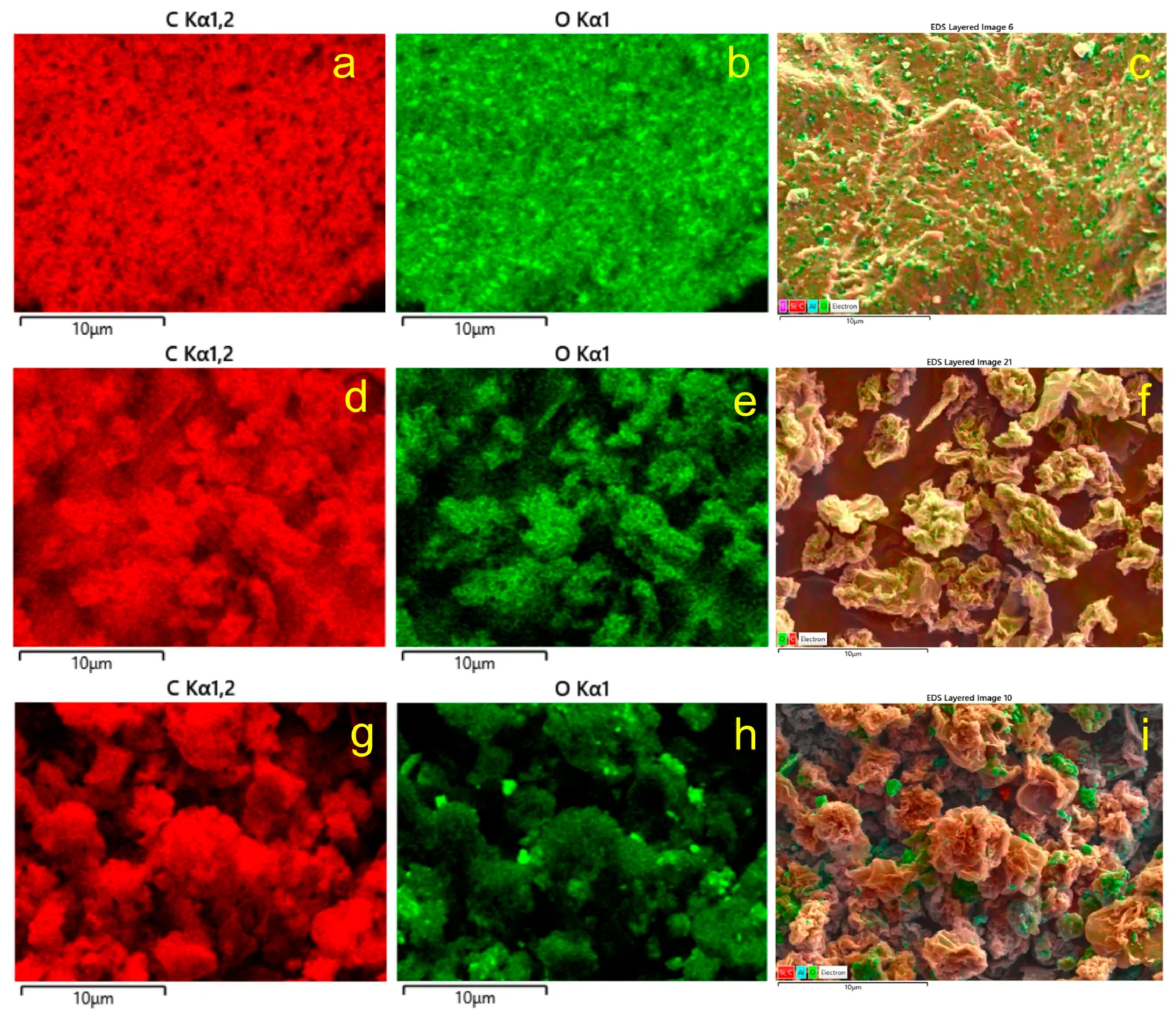
EDS mapping of the studied samples: (**a**–**c**) HA; (**d**–**f**) rGO; and (**g**–**i**) HA-rGO. For each sample, carbon and oxygen distribution maps and the combined elemental map are shown.

To examine the distribution of the main elements in the initial humic acid, reduced graphene oxide, and HA-rGO composite, energy-dispersive X-ray spectroscopy with elemental mapping was performed. The obtained results are presented in Figure 4.

According to EDS mapping, carbon and oxygen are present over the entire analyzed surface in all samples. For humic acid, a relatively uniform distribution of carbon and oxygen is observed, which is consistent with the high content of oxygen-containing fragments in the humic material structure [1,2,3,4].

Reduced graphene oxide is characterized by the predominance of the carbon component. The elemental distribution maps show a developed carbon framework with a less intense oxygen distribution, corresponding to partial removal of oxygen-containing groups during the reduction in graphene oxide [10,11,14,44].

The most informative results were obtained for the HA-rGO composite. The carbon and oxygen distribution maps show the presence of both elements over the entire studied surface. At the same time, no pronounced spatial separation of the components or isolated regions characteristic of individual phases is observed. The combined elemental map indicates the absence of pronounced spatial separation of the components within the analyzed region of the composite surface.

**Figure 5:**
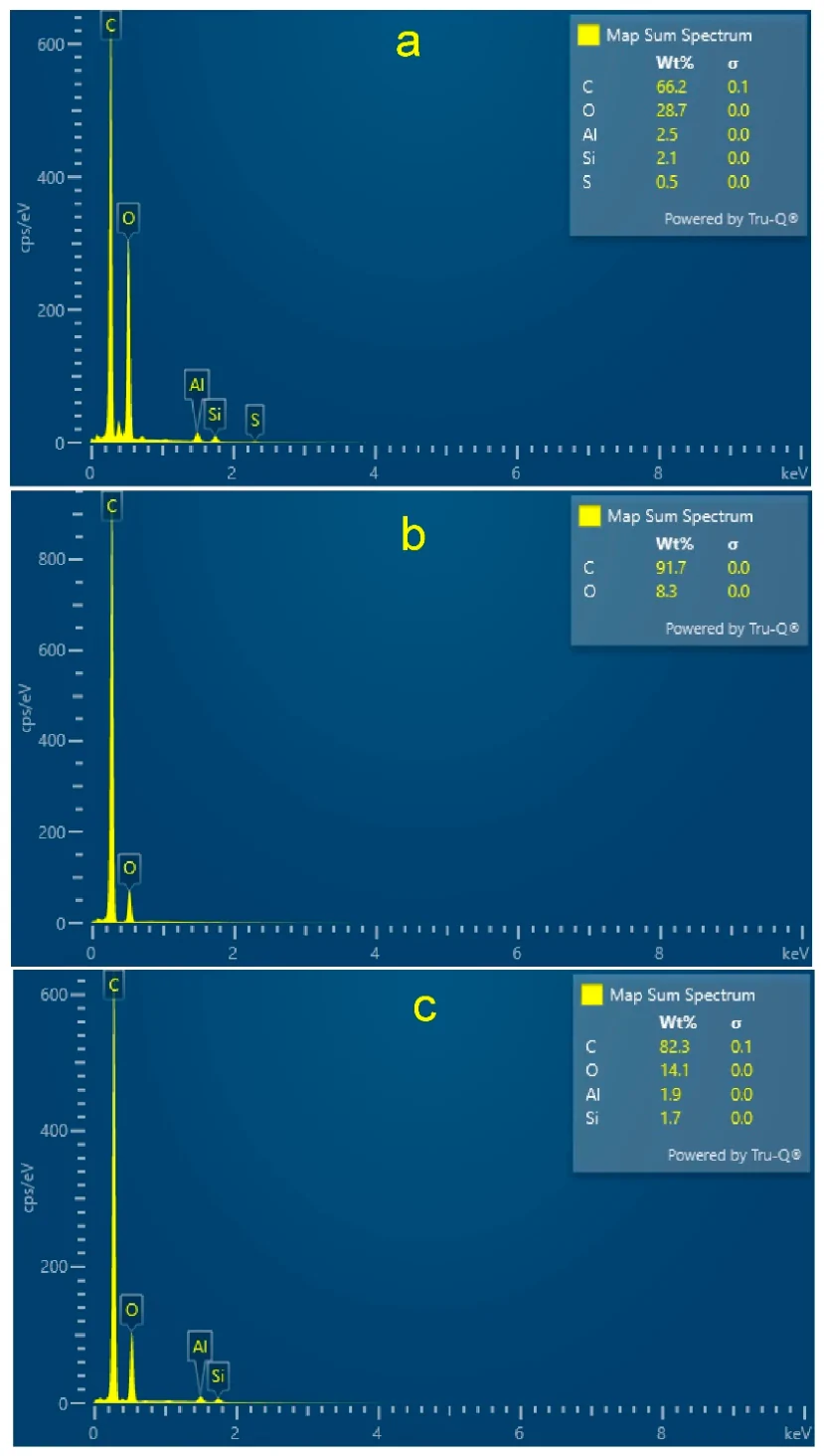
EDS spectra of the studied samples: (**a**) HA; (**b**) rGO; and (**c**) HA-rGO.

For a more detailed analysis of the elemental composition, EDS spectra were obtained for the initial humic acid, reduced graphene oxide, and synthesized HA-rGO composite (Figure 5). The spectra confirm the presence of the main elements identified by mapping and allow evaluation of the elemental-composition features of the samples.

Analysis of the EDS spectra shows that reduced graphene oxide has the highest carbon content and the lowest oxygen content, which is consistent with the features of its graphene-like structure. In the humic acid spectrum, in addition to carbon, a more pronounced oxygen contribution is observed due to the presence of oxygen-containing functional groups.

The HA-rGO composite is characterized by the simultaneous presence of significant amounts of carbon and oxygen. This result confirms the presence of oxygen on the composite surface after its formation. In addition, minor amounts of aluminum and silicon are detected in the spectra of HA and the composite, probably associated with the mineral component of the initial coal feedstock. Their contents are low and do not substantially affect the structure of the obtained material.

Thus, EDS mapping and energy-dispersive analysis confirm the presence of the main elements characteristic of the studied materials and indicate effective distribution of the components within the HA-rGO composite. The obtained data indicate a relatively uniform distribution of carbon and oxygen within the analyzed surface regions of the composite.

It should be noted that elemental analysis and EDS data cannot be directly compared quantitatively. Elemental analysis characterizes the average composition of the whole sample, whereas EDS reflects the local elemental composition of the analyzed surface region. Therefore, these methods were considered complementary in characterizing the studied materials [44,45].

### 3.6. Proposed Model of HA-rGO Composite Formation

**Figure 6:**
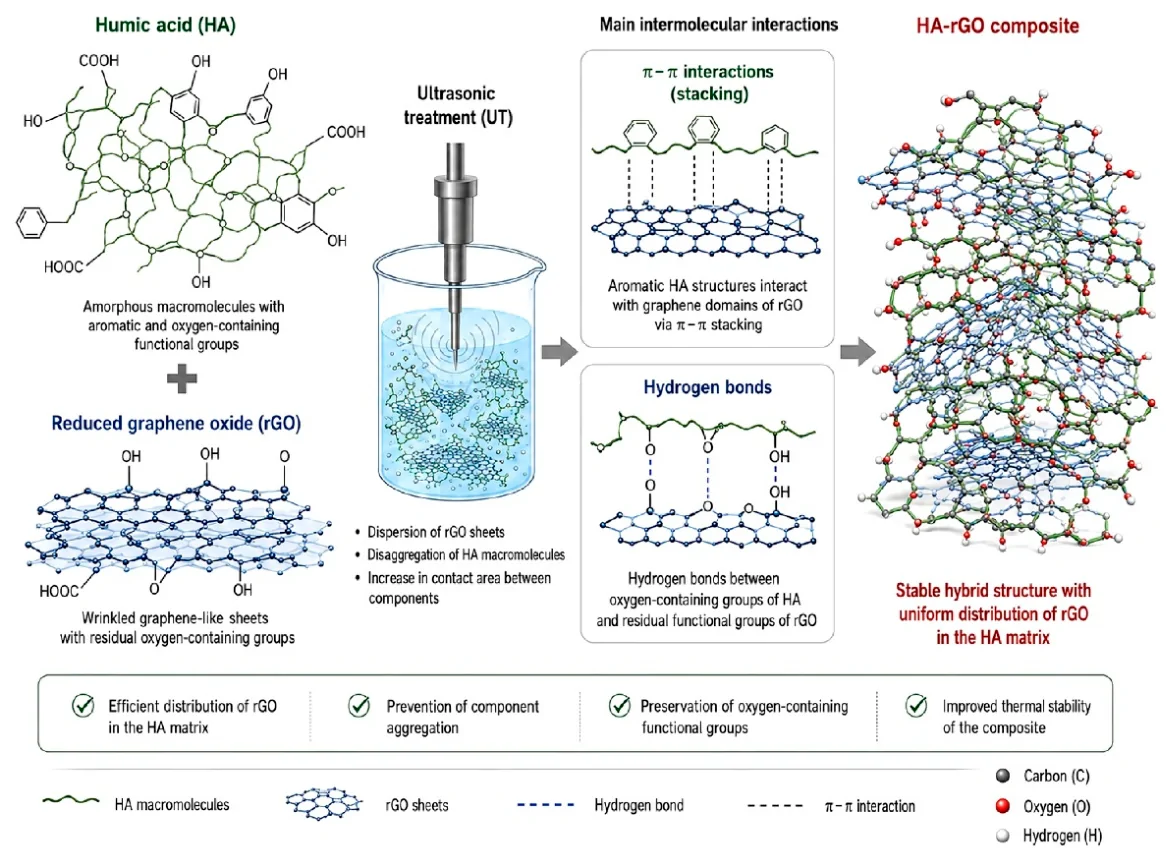
Proposed model of HA-rGO composite formation during ultrasonic treatment.

Based on the results of elemental analysis, FTIR spectroscopy, thermogravimetric analysis, scanning electron microscopy, and EDS mapping, a model for the formation of the composite based on humic acid and reduced graphene oxide was proposed (Figure 6).

The composite was synthesized in an alkaline medium under ultrasonic treatment of a suspension of humic acid and reduced graphene oxide. Under these conditions, dispersion of graphene sheets and simultaneous disaggregation of humic acid macromolecular associates may occur. Ultrasonic exposure increases the contact area between the components and improves the accessibility of functional groups for intermolecular interaction [34,35,36,37].

According to FTIR spectroscopy, after composite synthesis the main absorption bands characteristic of both humic acid and reduced graphene oxide are retained. At the same time, no new bands indicating the formation of additional covalent bonds are detected. This suggests that composite formation occurs mainly through non-covalent interactions between the components.

One of the most probable interaction types is π–π stacking between the aromatic fragments of humic acid and the graphene-like layers of reduced graphene oxide. The developed system of conjugated aromatic structures in both components creates favorable conditions for the formation of stable interplanar interactions [15,16,21].

Hydrogen bonds between the oxygen-containing functional groups of humic acid and residual hydroxyl, carboxyl, and epoxy groups on the surface of reduced graphene oxide may also contribute to stabilization of the composite structure. The combination of π–π interactions and hydrogen bonding can anchor humic macromolecules on the surface of graphene sheets and hinder their reaggregation [10,14,18].

Scanning electron microscopy shows a substantial change in the morphology of the material after synthesis. Unlike the initial components, the composite forms large wrinkled aggregates with complex spatial organization. The absence of clearly separated graphene sheets and dense humic acid aggregates in the examined SEM regions is consistent with substantial morphological integration of the components [44,45,46,47].

EDS mapping shows the presence of carbon and oxygen over the entire composite surface without pronounced spatial separation of the components. This result supports the proposed distribution of the components within the examined regions of the composite surface.

Thermogravimetric analysis provides additional evidence of structural interaction between the components. Compared with the initial humic acid, the composite exhibits higher thermal stability and a higher residual mass at 1000 °C. This may be related both to the presence of the thermally stable graphene component and to the formation of a more condensed carbon structure due to interfacial interactions between the components [20,42,43].

Taken together, the obtained data suggest that formation of the HA-rGO composite occurs mainly through a set of non-covalent interactions, including π–π stacking of aromatic structures and hydrogen bonding between the functional groups of the components. The result is a stable hybrid structure with altered surface morphology, increased thermal stability, and retention of a significant amount of oxygen-containing functional groups.

### 3.7. Preliminary Phenol Adsorption Assessment

Phenol adsorption was used as a preliminary functional assessment of the synthesized HA-rGO composite. This experiment was not intended to provide a complete technological evaluation of the material for wastewater treatment, but rather to establish a relationship between the structural-functional changes observed after composite formation and the ability of the material to interact with an aromatic organic pollutant.

The obtained adsorption data showed that the HA-rGO composite exhibited a higher adsorption capacity toward phenol than the initial humic acid and rGO. This behavior demonstrates that composite formation enhances phenol uptake under the investigated experimental conditions. The enhanced adsorption performance can be attributed to the combined contribution of oxygen-containing functional groups of humic acid and the aromatic carbon structure of rGO.

Carboxyl, phenolic, and hydroxyl groups present in humic acid may participate in hydrogen bonding with phenol molecules, whereas the graphene-like aromatic domains of rGO may promote π–π interactions with the aromatic ring of phenol. Therefore, the improved adsorption behavior of the HA-rGO composite is likely associated not only with the increased contribution of the carbon phase but also with the retention and accessibility of oxygen-containing functional sites.

**Figure 7:**
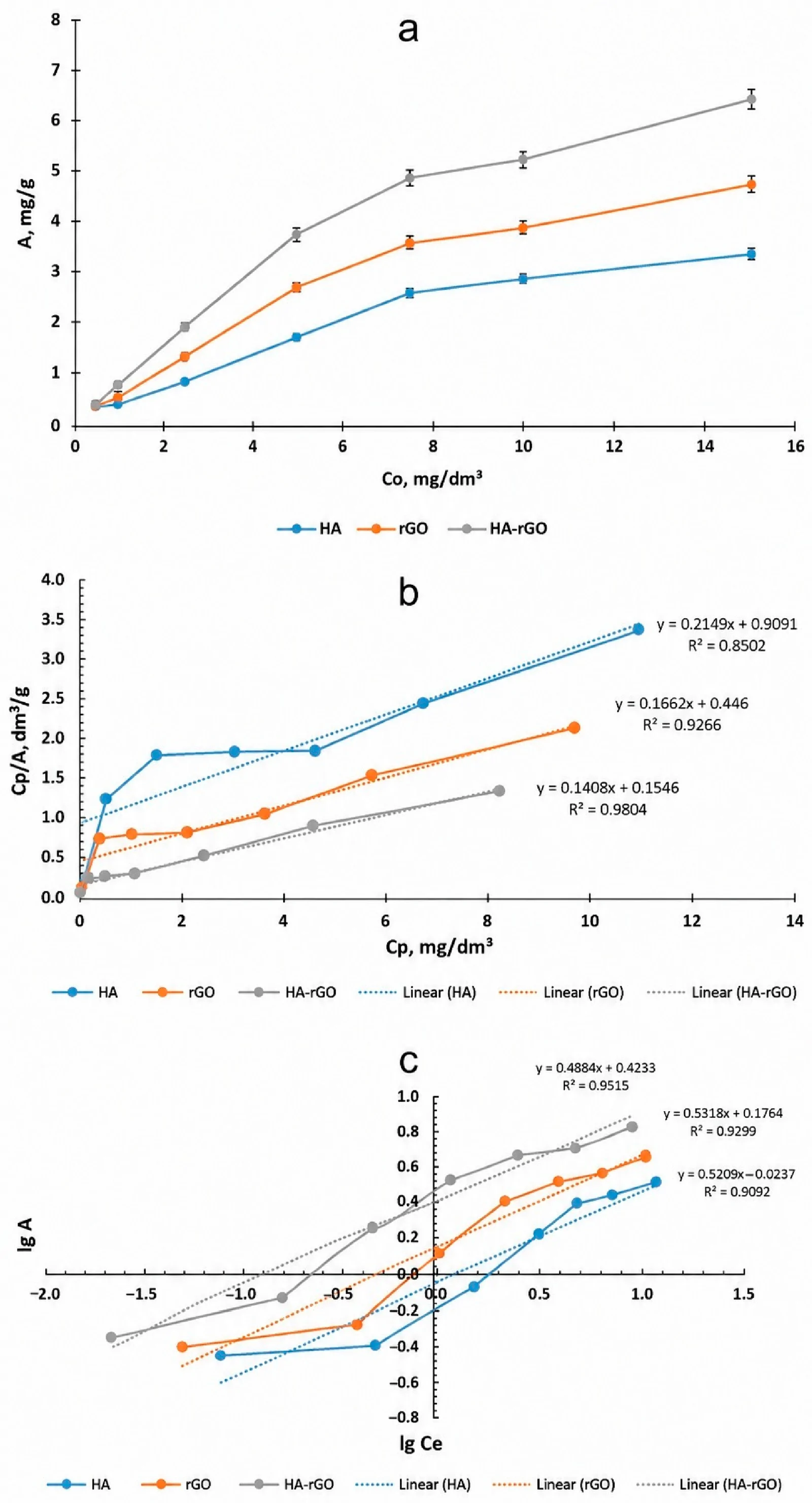
Phenol adsorption equilibrium data and linearized isotherm plots for HA, rGO, and HA–rGO: (**a**) experimental equilibrium adsorption isotherms; (**b**) linearized Langmuir plots; and (**c**) linearized Freundlich plots. Error bars in panel (**a**) represent the standard deviations of three independent measurements. The lines in panels (**b**,**c**) represent linear regression fits.

To quantitatively evaluate the agreement between the experimental equilibrium data and the theoretical adsorption models, the results were analyzed using the Langmuir and Freundlich isotherms. The experimental adsorption data and the corresponding linearized model plots are presented in Figure 7.

The calculated parameters of the Langmuir and Freundlich models are summarized in Table 3. The adsorption of phenol on HA was better described by the Freundlich model, for which the coefficient of determination was R^2^ = 0.9092, compared with R^2^ = 0.8502 for the Langmuir model. This result is consistent with the heterogeneous supramolecular structure of humic acid and the presence of adsorption sites with different interaction energies.

For rGO, the Langmuir and Freundlich models showed comparable agreement with the experimental data, with R^2^ values of 0.9266 and 0.9299, respectively. Therefore, neither model can be considered unambiguously predominant for rGO within the investigated concentration range.

In contrast, phenol adsorption on the HA–rGO composite was better described by the Langmuir model, with R^2^ = 0.9804, whereas the Freundlich model gave R^2^ = 0.9515. The maximum adsorption capacity calculated according to the Langmuir model was 7.10 mg/g for HA–rGO, compared with 6.02 mg/g for rGO and 4.65 mg/g for HA. The higher qm value obtained for the composite agrees with its experimentally observed enhanced affinity toward phenol.

The better agreement of the HA–rGO adsorption data with the Langmuir model indicates a more uniform occupation of the accessible adsorption sites within the investigated concentration range. Nevertheless, the model fit should be regarded primarily as a mathematical description of the equilibrium data and not as direct proof of a strictly homogeneous monolayer adsorption mechanism.

**Figure 8:**
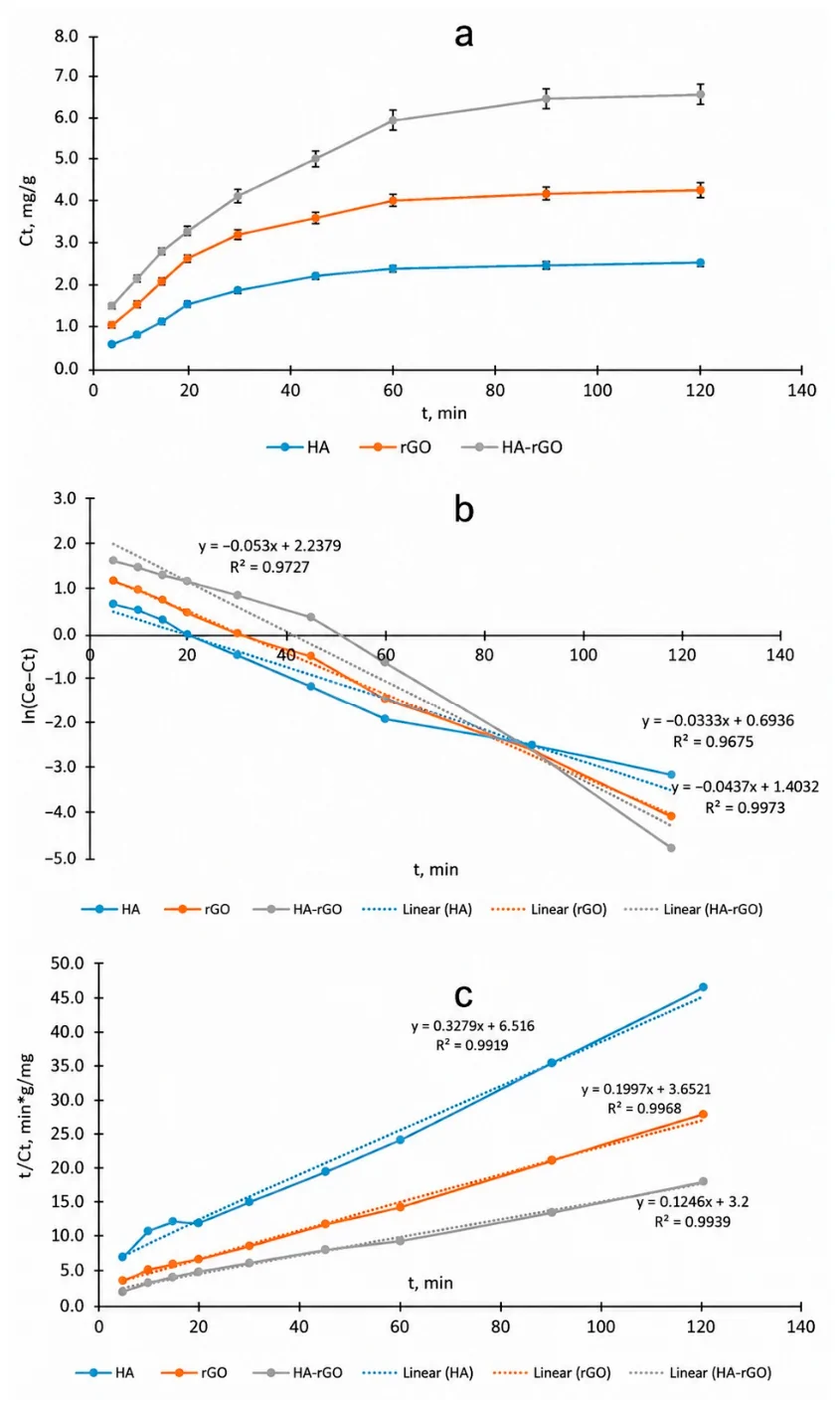
Phenol adsorption kinetics and linearized kinetic-model plots for HA, rGO, and HA–rGO at an initial phenol concentration of 7.5 mg/dm^3^: (**a**) experimental adsorption kinetics; (**b**) linearized pseudo-first-order plots; and (**c**) linearized pseudo-second-order plots. Error bars in panel (**a**) represent the standard deviations of three independent measurements. The fitted lines in panels (**b**,**c**) represent linear regression results.

To quantitatively evaluate the adsorption kinetics, the experimental data were analyzed using the pseudo-first-order and pseudo-second-order kinetic models. The experimental kinetic curves and the corresponding linearized model plots are presented in Figure 8.

The calculated kinetic parameters are summarized in Table 4. Phenol adsorption proceeded rapidly during the initial stage, followed by a gradual decrease in the adsorption rate as equilibrium was approached.

For HA, the pseudo-second-order model provided a better description of the experimental data, with R^2^ = 0.9919, compared with R^2^ = 0.9675 for the pseudo-first-order model. The pseudo-second-order model also showed better agreement for the HA–rGO composite, with an R^2^ value of 0.9939 compared with 0.9727 for the pseudo-first-order model.

For rGO, both models showed very high and nearly identical coefficients of determination. The pseudo-first-order model exhibited a marginally higher R^2^ value of 0.9973 compared with 0.9968 for the pseudo-second-order model. Moreover, the equilibrium adsorption capacity calculated using the pseudo-first-order model was closer to the experimental value for rGO.

Thus, the kinetic data for HA and HA–rGO were better described by the pseudo-second-order model, whereas both models provided a comparable fit for rGO. These models should be regarded as empirical descriptions of the adsorption-rate data and do not, by themselves, establish the chemical nature of adsorption or identify a unique rate-controlling mechanism.

The adsorption performance of HA–rGO was compared with selected adsorbents previously investigated for the removal or recovery of phenolic compounds (Table 5). Hydroxyapatite nanopowders exhibited a maximum phenol adsorption capacity of 10.33 mg/g; however, this value was obtained at an initial phenol concentration of 400 mg/L and a temperature of 60 °C [48]. Graphene was reported to adsorb up to 28.26 mg/g of phenol at an initial concentration of 50 mg/L and pH 6.3 [49]. These experimental conditions differ substantially from those used in the present study, particularly with respect to the initial phenol concentration. Therefore, direct comparison based exclusively on the maximum adsorption capacity should be made with caution.

The studies recommended by the reviewer also demonstrate that adsorption technologies can be applied to more structurally complex phenolic compounds [50,51]. Acrylic adsorbent resins have been used at pilot scale for the recovery and fractionation of apple polyphenols [52], whereas recoverable bismuth-based alginate materials have been applied in a combined adsorption–photodegradation process for gallic acid and 3,4,5-trimethoxybenzoic acid [53]. These systems differ from the present HA–rGO material in adsorbate structure, operating conditions and intended application and are therefore discussed as contextual examples rather than directly comparable adsorbents.

The adsorption capacity of HA–rGO is lower than the values reported for some highly porous graphene-based adsorbents [49]. However, the present composite was evaluated in a relatively low phenol concentration range of 0.5–15 mg/dm^3^ and was not specifically optimized for maximum adsorption capacity. Its principal feature is the combination of a humic matrix containing oxygen-bearing functional groups with the aromatic carbon structure of rGO. Accordingly, the present results should be regarded as a preliminary demonstration of enhanced phenol interaction rather than evidence of superiority over existing adsorbents.

The proposed interpretation of phenol adsorption is based on the combined results of elemental analysis, functional-group titration, FTIR spectroscopy, SEM/EDS observations and adsorption experiments. Hydrogen bonding and π–π interactions are therefore considered plausible contributions rather than directly demonstrated elementary mechanisms. Because XPS, BET surface-area measurements, pore-size-distribution analysis and determination of the point of zero charge were not performed, the chemical states of the surface groups, the quantitative contribution of textural properties and the role of surface charge could not be established directly. Consequently, the proposed adsorption interactions should be regarded as a preliminary mechanistic interpretation requiring further experimental verification.

Overall, the preliminary phenol adsorption assessment confirms the functional relevance of the structural changes induced by HA-rGO composite formation. The combination of oxygen-containing functional groups of humic acid and the aromatic carbon structure of rGO provides more effective interaction with phenol, indicating the potential of the obtained composite for the adsorption of low-concentration aromatic organic pollutants.

## 4. Conclusions

In this work, a humic acid/reduced graphene oxide composite was synthesized by ultrasonic treatment. The incorporation of reduced graphene oxide increased the carbon content from 47.34 to 55.61 wt.% and decreased the oxygen content from 48.31 to 40.79 wt.%, indicating the formation of a more carbon-rich hybrid material. At the same time, the total content of carboxyl and phenolic hydroxyl groups increased to 5.47 mmol/g, confirming the retention and increased accessibility of oxygen-containing functional groups in the composite structure.

FTIR spectroscopy showed that the main structural fragments of humic acid and reduced graphene oxide were retained after composite synthesis. The absence of new characteristic absorption bands suggests that composite formation occurs mainly through non-covalent interactions, including π–π interactions between aromatic structures and hydrogen bonding between oxygen-containing functional groups.

Thermogravimetric analysis demonstrated improved thermal stability of the HA-rGO composite compared with the initial humic acid. The increase in residual mass at 1000 °C from 59.21 to 70.03% indicates the formation of a more thermally stable carbon-containing structure. SEM and EDS analyses confirmed substantial changes in surface morphology, the formation of a developed wrinkled structure, and the distribution of carbon and oxygen over the composite surface without pronounced phase separation.

The preliminary phenol adsorption assessment showed that the HA-rGO composite exhibited a higher adsorption capacity than the initial humic acid and rGO. This improvement can be associated with the combined contribution of oxygen-containing functional groups and the aromatic carbon structure of rGO, which may facilitate hydrogen bonding and π–π interactions with phenol molecules. The adsorption and kinetic results confirm the functional relevance of the structural changes induced by composite formation. Unlike previously reported studies focused primarily on carbonized HA–GO derivatives or the conversion of humic acid into graphene-like materials, the present study characterizes a non-carbonized HA–rGO composite in which the functional humic matrix is retained.

Thus, the obtained HA-rGO composite is a promising carbon-containing material with enhanced thermal stability, developed surface morphology, accessible oxygen-containing functional groups, and improved interaction with low-concentration aromatic organic pollutants. Further studies involving regeneration, competing pollutants, and real water matrices are required for a complete technological evaluation of its adsorption performance.

## Figures and Tables

**Table 1:** Elemental composition, content of oxygen-containing functional groups, and yield of the studied samples.

Sample	C, wt.%	H, wt.%	O, wt.%	Σ(COOH + OH), mmol/g	O/C (Atomic)	Yield, %
HA	47.34 ± 0.2	4.35 ± 0.2	48.31 ± 0.2	5.00 ± 0.2	0.77	70.05
rGO	67.49 ± 0.2	0.57 ± 0.2	31.94 ± 0.2	3.90 ± 0.2	0.36	–
HA-rGO	55.61 ± 0.2	3.60 ± 0.2	40.79 ± 0.2	5.47 ± 0.2	0.73	87.13

**Table 2:** Mass losses of the studied samples in different temperature intervals according to TGA.

Sample	30–150 °C, %	150–450 °C, %	450–700 °C, %	700–1000 °C, %	Residual Mass at 1000 °C, %
HA	3.00	12.15	9.19	1.90	59.21
HA-rGO	0.78	9.58	7.13	2.06	70.03
rGO	1.00	4.41	4.37	2.57	84.23

**Table 3:** Parameters of the Langmuir and Freundlich isotherm models for phenol adsorption.

Sample	q_m_, mg/g	K_L_, dm^3^/mg	R^2^, Langmuir	K_F_, (mg/g)(dm^3^/mg)^1/n^	n	R^2^, Freundlich
HA	4.6540	0.2363	0.8502	0.9469	1.9199	0.9092
rGO	6.0165	0.3726	0.9266	1.5011	1.8805	0.9299
HA-rGO	7.1018	0.9110	0.9804	2.6505	2.0476	0.9515

**Table 4:** Calculated parameters of the pseudo-first-order and pseudo-second-order kinetic models for phenol adsorption on the studied materials.

Sample	q_e,exp_, mg/g	q_e,cal_, mg/g	k_1_, min^−1^	R^2^, PFO	q_e,cal_, mg/g	k_2_, g·mg^−1^·min^−1^	R^2^, PSO
HA	2.60	2.0009	0.0333	0.9675	3.0498	0.0165	0.9919
rGO	4.28	4.0682	0.0437	0.9973	5.0079	0.0109	0.9968
HA-rGO	6.50	9.3735	0.0530	0.9727	8.0239	0.0049	0.9939

**Table 5:** Comparison of the phenol adsorption performance of HA–rGO with selected previously reported adsorbents.

Adsorbent	Adsorbate	Maximum Adsorption Capacity, mg/g	Main Conditions	Reference
HA-rGO	Phenol	7.10	C_0_ = 0.5–15 mg/dm^3^, 25 °C	This study
Hydroxyapatite nanopowder	Phenol	10.33	C_0_ = 400 mg/L, pH 6.4, 60 °C	Lin et al. [48]
Graphene	Phenol	28.26	C_0_ = 50 mg/L, pH 6.3, ~12 °C	Li et al. [49]
HA–carbon hybrid material	Phenol	-	Adsorption dependent on pH, concentration and hybrid composition	Li et al. [49]

## Data Availability

The original contributions presented in this study are included in the article. Further inquiries can be directed to the corresponding author.

## Abbreviations

The following abbreviations are used in this manuscript:

HA	Humic acid
rGO	Reduced graphene oxide
HA-rGO	Humic acid/reduced graphene oxide composite
FTIR	Fourier transform infrared spectroscopy
TGA	Thermogravimetric analysis
TG	Thermogravimetry
DTG	Derivative thermogravimetry
DTA	Differential thermal analysis
SEM	Scanning electron microscopy
EDS	Energy-dispersive X-ray spectroscopy
O/C	Atomic oxygen-to-carbon ratio
